# A Systematic Review of Interventions to Address Accent-Related Communication Problems in Healthcare

**DOI:** 10.31486/toj.19.0028

**Published:** 2019

**Authors:** Yulong Gu, Amee P. Shah

**Affiliations:** School of Health Sciences, Stockton University, Galloway, NJ

**Keywords:** *Communication barriers*, *delivery of health care*, *education–medical*, *speech production measurement*, *systematic review*

## Abstract

**Background:** Linguistic diversity in terms of speech, accent, and dialect can present a barrier to communication in healthcare. This review synthesizes current evidence on the effectiveness of interventions that target accent- or dialect-related communication problems in healthcare.

**Methods:** Eligible studies were identified through a search of literature databases in 2018 using medical subject heading (MeSH) terms and additional keywords. Articles were screened for eligibility according to predetermined inclusion criteria. Because of the heterogeneity of the studies, effect data were analyzed using narrative synthesis.

**Results:** Twenty-six studies (n=964) were included, reporting a range of interventions that provided accent modification training, role play or simulated patient consultation training, general language and consultation skills training, web-based training, and cultural competence training. Most studies (24 of the 26) indicated some benefits of the accent elements in the interventions based on objective or subjective measures. The key benefits were improvements in speech production, communication competence, and academic/clinical performance, as well as perceptual changes.

**Conclusion:** Notwithstanding the low research quality and lack of standardized measures in the included studies, this review establishes an evidence base for implementing accent-related communication improvement strategies in healthcare. Large randomized controlled studies would be helpful to strengthen this evidence base.

## INTRODUCTION

The quality of communication in healthcare has a significant impact on patient-clinician relationships and patient satisfaction,^1^ patient safety,^2^ health outcomes,^3^ care team collaboration,^4^ and professional liability.^5^ Communication quality problems related to linguistic diversity in terms of accent and dialect are a growing issue in healthcare given the demographic change in the general population and global migration of clinicians. For instance, the United States is the largest importer of nurses, with approximately 219,000 registered nurses who were trained overseas.^6^ More than one-third of the United Kingdom National Health Service medical workforce was trained overseas,^7^ and one-third of the doctors working in Australia were trained overseas.^8^ In addition, the growing cultural heterogeneity in patient populations resulting from immigration has been noted in the United States and Europe.^9,10^ The globalization of the healthcare workforce and patient populations presents a challenge for both clinician-patient communication and clinician-clinician communication. Part of this challenge is to achieve speech clarity and comprehension when speakers with different accents or dialects participate in a verbal conversation. For understanding to occur, the speaker must deliver intelligible speech, and the listener must recognize and understand unfamiliar speech.

According to the American Speech-Language-Hearing Association, an accent is a phonetic trait from a person's original language that is carried over into a second language, while dialect is a broader term that may include pronunciation differences, semantic differences of word choice, and grammatical differences (including sentence word order) that denote regional areas or social groups.^11,12^ Although accent and dialect are natural characteristics of speech, they can present a barrier to effective communication in healthcare where a variety of accents and dialects are present.^13,14^ Immigrant health professionals’ and patients’ strong accents can reduce speech intelligibility. Also often observed during patient consultations is the use of colloquial expressions associated with a local dialect that are incomprehensible to those unfamiliar with the expressions. Research shows that accented speech slows listeners’ processing time.^15^ In the healthcare setting, accents and dialects have been associated with perceptions of lower clinical competency in clinician-clinician communications^16^ and clinician-patient communications, with shorter consultations,^17^ with lower care quality,^18^ and with less trust for the healthcare provider.^19^

A targeted strategy to address the communication problems in healthcare related to accent and/or dialect is accent modification training programs, often conducted among health students such as medical residents^20^ and nursing students.^21,22^ Another potential strategy, validated in the laboratory setting, focuses on accent adaptation through exposure to systematic variability in accented speech, via either listening exercises^23^ or listening and imitating accented speech.^24^ Other strategies include using tools and technologies to assist with health communication, such as giving patients a printout of their results or a written explanation to aid patient consultation with accented general practitioners.^14^ However, little is known about the impact of these strategies in the healthcare setting. A 2016 systematic review of communication training programs for culturally and linguistically diverse health students concluded that objective evidence of improved skills or performance posttraining is limited.^25^ In this review, we expanded the scope of the 2016 review^25^ to examine interventions that address accent- and dialect-related communication problems in all healthcare contexts, including health education programs and healthcare delivery systems. The intervention subjects include culturally and linguistically diverse health students and professionals (such as immigrant healthcare professionals), professionals who are minorities or for whom English is their second language (ESL), international medical graduates, and international nursing graduates. Our goal was to synthesize the evidence on the effectiveness of interventions that target communication problems related to accent or dialect in healthcare.

## METHODS

To identify scientific evaluations of accent- or dialect-related interventions in healthcare, we systematically searched and selected relevant studies^26,27^ and included both quantitative and qualitative studies in this review, as findings from all types of studies may contribute to understanding the effectiveness of interventions.^28^ Before undertaking the review, we developed a review protocol according to the Preferred Reporting Items for Systematic Reviews and Meta-Analyses (PRISMA) guideline.^29^ The protocol defined the literature search strategy (Table 1), study eligibility criteria (Table 2), study selection procedure, quality of evidence criteria, and data collection and analysis methods.

**Table 1.** Literature Search Strategy

Boolean Operator	Search Terms
	(accent OR dialect)
AND	(*communication* OR “*communication barriers*” OR “clinical communication” OR “communication skills” OR “communication competency” OR “cross-cultural communication” OR “professional communication” OR “culturally and linguistically diverse” OR CALD OR bilingual OR multilingual OR “English as a second language” OR ESL OR “limited English proficiency” OR *consultation* OR *speech* OR “clear speech” OR speak OR pronunciation OR intelligibility OR *comprehension* OR *understanding* OR *perception* OR “*speech perception*” OR stereotype)
AND	(*health* OR *healthcare* OR *medicine* OR medical OR clinic OR clinical OR “*health occupations*” OR “*foreign professional personnel*” OR “*foreign medical graduates*” OR “international medical graduate” OR IMG OR clinician OR *physicians* OR doctor OR *nurses* OR *nursing* OR *pharmacists* OR *pharmacy* OR *dentists* OR dental OR “*allied health professional*” OR “*occupational therapists”* OR OT OR “*occupational therapy”* OR “*physical therapists”* OR PT OR “physical therapy” OR “speech-language pathologist” OR SP OR SLP OR “*speech-language pathology”* OR *caregivers* OR *patients* OR “healthcare consumers”)
AND	(“accent modification” OR “accent reduction” OR “accent management” OR “accent adaptation” OR “accent processing” OR “accent identification” OR “accent detection” OR “listening exercise” OR “accent listening” OR “vocal imitation” OR intervention OR *therapy* OR *treatment* OR remediation OR program OR training OR *education* OR *teaching* OR strategy OR initiative OR support OR *technology* OR tool)
AND	(evaluate OR assess OR outcome OR results OR measure OR *standards* OR test OR “English language ability test” OR “English proficiency test” OR screening OR effect OR impact OR efficacy OR cost-effectiveness OR retention OR attrition OR clarity OR feedback)

Note: The search terms in italic font are medical subject heading (MeSH) terms.

CALD, culturally and linguistically diverse; ESL, English as a second language; IMG, international medical graduate; OT, occupational therapy; PT, physical therapy; SLP, *speech-language pathology;* SP, speech-language pathologist.

**Table 2.** Study Eligibility and Exclusion Criteria

Inclusion Criteria	Exclusion Criteria
1. Studies were published from 1/1/1990 to 7/10/2018.	1. Studies were published prior to 1990.
2. Studies are written in English.	2. Studies are written in a language other than English.
3. The publication is peer-reviewed.	3. The publication is not peer-reviewed or the information is from nonscholarly sources such as newspaper articles.
4. Full text is available.	4. Full text is not available, only abstracts.
5. Studies have an adequate description of an accent intervention or strategy in a healthcare setting or a health education setting.a. Population: Study participants are healthcare professionals, health students, health educators, or patients/families/caregivers.b. Intervention: Programs (including tools or training) are provided to support clinicians/patients for the purpose of improving health communications, and one of the intervention's goals, explicitly or implicitly, is to mitigate the negative impact on communication quality due to accent or dialect. Such interventions may have a training element on pronunciation, tone, stress, colloquialism, or listening comprehension for culturally and linguistically diverse healthcare professionals/students.c. Comparison: Studies evaluated intervention effects by comparing with preintervention or no intervention. Other randomized/quasiexperimental designs and qualitative feedback by participants or trainers on communication skills improvement or on other intervention effects are acceptable.d. Outcome: Studies have objective and/or subjective measures to assess impact, efficacy, or cost-effectiveness of the intervention.e. Setting: Studies are conducted in healthcare delivery systems, community-based health programs, or health education programs.	5. Studies have an insufficient description of the intervention, the intervention objectives do not include accent- or dialect-related communication improvement, or the intervention was not implemented in a healthcare delivery or health education setting.a. Population: Patient participants have specific communication disorders.b. Intervention: Treatment interventions are for specific communication disorders or interventions to improve English proficiency without addressing accent- or dialect-related issues. Studies that examined the effect of using interpreters, translators, or transcribers (human or technology-based) in healthcare delivery were also excluded.c. Comparison: Studies have no comparison and no demonstration of intervention effects but only report on personal experiences as a status snapshot (eg, a single survey on communication issues).d. Outcome: Studies do not have any specific outcome measures.e. Setting: Studies are conducted in preschool, primary, secondary, or tertiary classrooms that are not part of a health education program or in English as a second language training classrooms for non-healthcare professionals/students (eg, accent modification training programs for business school students). Studies to evaluate efficacy of accent interventions in the laboratory setting (eg, speech science research in a laboratory) were also excluded.
6. Studies have quantitative or qualitative outcome measures that rigorously assess the effect of the intervention. The direct intervention effect on the individual and organization and the indirect effect (eg, on patient outcome) are of interest in this review. The intervention effect in terms of communication quality, clinical performance, health outcome, and cognitive or behavioral change are to be considered.	6. No specific outcome measures are reported, or the effect data are not analyzed using scientific methods.
7. English is the dominant language for communication in the clinical or academic setting.	7. English is not the dominant language for communication in the study setting.
8. All study designs used to evaluate an individual intervention (eg, randomized controlled trials, other comparative studies, surveys, descriptive studies, etc) were included if they fit the criteria above.	8. Expert opinions and review of studies were excluded.

### Literature Search Strategy

Eligible studies published between 1990 and 2018 were identified through a search of literature databases using medical subject heading (MeSH) terms and additional search terms relevant to the research topic. The databases searched included MEDLINE (via PubMed), Cochrane Central Register of Controlled Trials (via Ovid), Embase (via Ovid), Cumulative Index to Nursing and Allied Health Literature (via EBSCOhost), PsycINFO (via EBSCOhost), Education Resources Information Center (via EBSCOhost), Linguistics Database (via ProQuest), and ScienceDirect. To retrieve further possible articles, we searched the reference lists in the examined studies and in relevant systematic reviews, conducted a hand search of relevant authors and journals, and searched Google Scholar for grey literature such as unpublished studies and dissertations, conference proceedings, and university websites.

### Study Selection Procedure

The authors independently reviewed the titles and/or abstracts of all retrieved articles for eligibility according to the study eligibility criteria (Table 2). We discussed and reconciled discrepancies in screening decisions on 5 abstracts; none of the 5 was included in full-text screening. We then retrieved and reviewed the full text of the remaining studies using the same procedure: independent reviews by the authors followed by discussions until the authors agreed upon the selection decisions.

### Criteria for the Quality of Evidence

To assess the validity of included studies, we examined each individual study in terms of the level of evidence,^30^ the quality of evidence,^31^ the overall research quality (considering the research method, reported measures, and study rigor), and the risk of bias.^32^ Taking into account the wide range of research designs and outcome measures used in accent studies, we defined the following criteria based on existing quality appraisal tools and clinical expertise to evaluate the quality of accent intervention studies:

- High quality: High-level evidence (eg, randomized controlled trial [RCT], cohort, and case-control studies) with low risk of bias and statistical significance tested of objective measures related to communication quality (including flow-on effects such as patient outcome)
- Moderate quality: RCT, cohort, or controlled studies with low risk of bias but with no statistical testing of objective measures (eg, reporting only subjective measures such as qualitative feedback)
- Low quality: Uncontrolled studies (eg, case series) or poor-quality cohort control studies with significant limitations in scientific rigor, result generalizability, or reproducibility

### Data Collection and Analysis Procedures

Data from the eligible studies were extracted and recorded in Microsoft Excel. Multiple reports of one study were collated into a single data point. Extracted data items from each study included the following:

- Country where the study was conducted
- Study design
- Study quality evaluated as described in the Criteria for the Quality of Evidence section
- Type and number of intervention participants (health student, healthcare professional, or patient)
- Content, structure, duration, and delivery form of the accent or dialect intervention (only the intervention elements relevant to accent or dialect topics were included)
- Objective (standardized assessment tools such as established language tests and validated questionnaire surveys and nonstandardized measures based on a theoretical framework or on consistent rubrics such as intelligibility test grades assessed by the trainer) and subjective (qualitative feedback and self-rated competence without a rubric) outcome measures (pretest and posttest, if available) relevant to the short- and long-term effects of the intervention
- Study results

We planned both a meta-analysis and a narrative synthesis of the data. However, we found substantial heterogeneity among the studies and a range of nonstandardized measures used to assess the intervention effects. As a result, meta-analysis was infeasible, and the narrative synthesis method was used to analyze the data.

## RESULTS

We reviewed a total of 729 abstracts; 57 studies met the inclusion criteria and underwent full-text review. Among these, 31 studies were excluded (Figure). Of the 26 studies included in this review, 15 were conducted in the United States, 9 in Australia, and 2 in the United Kingdom. One of the 26 studies was a controlled before-after study, 22 were case series, and 3 were case studies.

**Figure.**
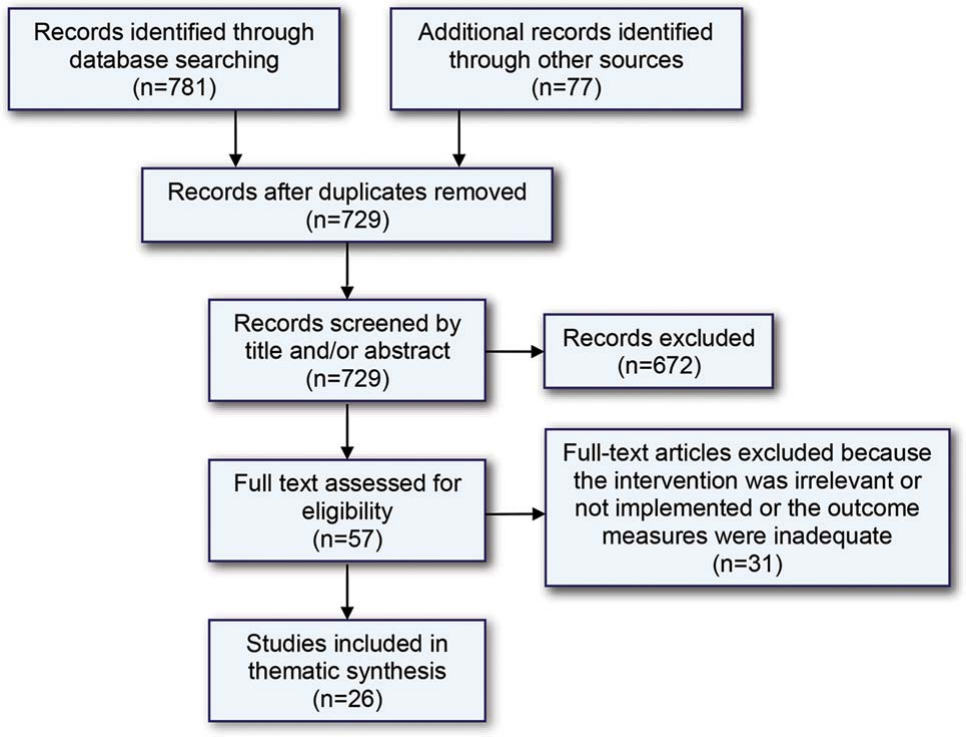
**Flowchart of search and selection of studies for review.**

### Characteristics of Accent Interventions

The studies included in this review described several accent intervention strategies:

- Accent reduction training, often led by a speech-language pathologist (SLP)^21,22,33-38^
- Role play or simulated patient consultation training^7,39-48^
- General language and consultation skill training^49-52^
- Internet-based education^53,54^
- Cultural competence training^55^

While each study involved communication skills training, all had distinctive program content, structure, and duration (ranging from 1 session to 2 semesters). Only 3 studies mentioned that the interventions were based on established accent interventions; one study^33^ was based on the Compton Pronouncing English as a Second Language Program,^56^ another study^37^ used the Comprehensive Assessment of Accentedness and Intelligibility model and method,^57^ and the third study had expanded the trainer's own accent reduction course regularly taught at a university.^38,58^ A range of linguistic elements related to accent or dialect were taught in the included studies: segmental production (consonants and vowels), phonological and prosodic rules, colloquial/idiom/dialect language, vocabulary and medical terminology, grammatical features, nonverbal/body language/pragmatic features to supplement speech understanding, auditory discrimination, and listening skills. Many studies targeted specific populations such as undergraduate and postgraduate culturally and linguistically diverse health students, international medical graduates, and culturally and linguistically diverse/ESL practicing health professionals. In the delivery of accent interventions, the majority of included studies (n=17, 65%) engaged a language expert, such as an SLP or English teacher. Among these 17 programs, 9 (often accent modification training programs) employed a language expert as the sole instructor,^21,33,34,36-38,45,49,50^ while 8 used a multidisciplinary teaching team that included both a language teacher and a clinical teacher.^7,22,41-44,47,48^ Study details are provided in Table 3.

**Table 3.** Summary of Accent- and Dialect-Related Intervention Elements and the Intervention Effects in the Included Studies

	Study	Participants		Accent Intervention		Objective Instruments		Subjective Measures		
Study	Quality	Type	n	Content/Structure	Duration	Pretest	Posttest	Pretest	Posttest	Effect Results
Yahes and Dunn, 1996^48^	Low	INGs	30	Weekly 2-hour sessions led by SLP and nursing educator. Content: pronunciation, accent remediation, phonetics.	12 weeks	None	None	None	Trainee feedbackClinical supervisor feedback	Anecdotal comments on increased job satisfaction, fewer communication-related complaints from physicians and staff, improved collegiality among staff, and decreased overall number of incident reports.
Chur-Hansen and Barrett, 1996^51^	Low	ESL medical students	44	Elective supplementary course on colloquial expressions. Structure: 2 tutorials + assignment to collect colloquial expression examples. Topic: colloquialism.	1 semester	None	None	None	Student course evaluation questionnaire	Students reported they learned informal language.
Chur-Hansen, 1999^52^	Low	ESL medical students	87	Part of weekly elective supplementary tutorials adjunct to Doctor, Patient and Society course. Accent topics: vocabulary and colloquial language.	2 semesters	None	Doctor, Patient and Society course grades	None	Student feedback	No conclusive benefit or harm. Tutorial attendees performed less well than nonattendees in the course, but the author stated, “without the program, the disparity in performance may have been much greater and the fail rates far more substantial.” Students reported the course as useful teaching support.
Symes et al, 2002^22^	Low	Nursing students	32	Part of a required 3-credit course, Student Success Program, led by an SLP and a nursing teacher; 15-hour accent modification + 2 4-hour periods of nurse shadowing + 4 oral presentations. Topics: medical and general vocabulary.	2 semesters	Nurse Entrance Test reading comprehension test	Student retention ratesAcademic and clinical pass rates	None	Trainee feedbackFaculty feedback	Some students reported more confidence about talking to patients and staff as a result of the course.
Bosher and Smalkoski, 2002^39^	Low	ESL health students	18	Part of a required 2-credit course that meets once a week for 2 hours. Course structure: lecture, discussion, reading, journal entry, student presentation, 9-10 role plays per student. Topic: paralinguistic features of communication (eg, tone, volume, and rate of speech).	14 weeks	Combined English Language Skills Assessment + Comprehensive English Language Test *or* University of Michigan English placement test *or* University of Michigan English Language Proficiency test	3 instructor-graded role-play scenariosFinal written exam	Student questionnaire (needs assessment)	Students’ course evaluation surveysAnecdotal comments by instructor and students	Anecdotal student success stories about improved communication skills. Positive responses on course usefulness, particularly role plays.
Guhde, 2003^35^	Low	ESL nursing student	1	Weekly 1-hour one-on-one tutoring by a nurse tutor. Topics: listening, speaking and pronunciation, and medical terminology.	10 weeks	Listening and note-taking test	Listening and note-taking test	None	Trainee feedback	Increased accuracy in note-taking.
Satter et al, 2005^55^	Low	Telephone survey interviewers	N/A	Training materials + in-person training. Topics: American Indian and Alaska Natives’ cultural and linguistic issues; reservation dialect English, long pauses, slow speaking, “walking on words,” and tribal heritage.	1 session	None	California Health Interview Survey response rate	None	Trainee feedbackTrainer observation	The health survey response rate in the target population is high; trainees reported training was helpful; trainer observed that trainees felt sensitive, connected, and responsible to the community and were motivated to do their best.
San Miguel et al, 2006^45^	Low	ESL nursing students	15	4-hour sessions per day for a total of 20 hours led by a language teacher. Topics: small-talk, formulaic expressions, vocabulary, grammar, and colloquial language/switching register.	5 weeks	None	Students’ clinical grades at the end of the next clinical placement	Student focus group on needsFacilitators’ written comments on students’ clinical performance	Student written feedback surveyStudent focus groupFacilitator focus group and interview	Improved communication skills and increased confidence, resulting in a more positive clinical experience for the majority of students.
Parkhurst, 2007^44^	Low	ESL pharmacy students	25	3-credit elective course. Structure: role plays, group practice, and presentations. Content: pronunciation, stress and intonation, register, grammar, medical terminology, and listening comprehension.	1 semester	Michigan Test of English ProficiencyOral summary examinationClass oral presentation	Exit examination using a 4-level holistic assessment rubric. Students were graded by 2 pharmacy faculty members.	None	Students’ course evaluation form	Improved oral skills (eg, intelligibility, stress, intonation, and vocabulary) and listening comprehension.
Seibold et al, 2007^46^	Low	CALD nursing students	20	Semester 1: fortnightly group meetings; Semester 2: group and individual meetings with academic mentors. Topics: colloquial language and pronunciation.	2 semesters	None	None	Course entry questionnaire (needs assessment)	Trainee focus groupTrainee exit questionnaireTrainee feedback questionnaire (3 months post-program)	The course helped develop oral and written communication skills.
Chiang and Crickmore, 2009^41^	Low	CALD nursing students (postgraduate)	N/A	Part of a full transition course led by language and clinical teachers, including 4-hour English instruction, 4-hour communication workshops, 2-hour clinical lab with simulated patients, and 32-hour clinical placement. Topics: social and colloquial language.	1 semester	None	None	None	Student interviewsTeacher feedback	No conclusive benefit or harm, but students reported that the course was useful and that it was helpful to learn about communication skills.
Shah, 2010^37^	Low	ESL anesthesiologist	1	Weekly 1-hour one-on-one accent modification therapy led by an SLP tailored to trainee needs based on diagnostic-based communication numeric scores. Topics: consonants, vowels, prosody, and suprasegmentals.	8 weeks	Comprehensive Assessment of Accentedness and Intelligibility	Comprehensive Assessment of Accentedness and Intelligibility	Language background questionnaire	Intelligibility assessed by SLP and 3 untrained listenersPretherapy and posttherapy audio comparisonsTrainee exit interview	Improvement in intelligibility, intonation, stress, and vowel and consonant production. Improved comfort and confidence with patients and presentation. Program satisfaction.
Boughton et al, 2010^40^	Low	CALD nursing students (postgraduate)	17	Part of CALD support program, led by 3 nursing faculty members. Five-hour introductory workshop + 9 1.5-hour sessions. Topic: colloquial language.	1 semester	None	None	None	Student interviews	Learning about effective communication and colloquial language enabled students to better understand various colloquialisms used by patients.
Cross and Smalldridge, 2011^7^	Low	IMGs	17	3 sessions (2 half-days + 1 full-day) with feedback by clinical and language teachers. Content: adjustments to accents and tone of voice. Structure: presentation, workshops, practice.	N/A	None	None	Formal feedback from supervising consultants	Trainee feedbackTrainer feedback	Improved communication skills.
Kokkinn and Stupans, 2011^43^	Low	ESL pharmacy students	82	Weekly 2-hour classes led by clinical and language specialists. Content: terminology, colloquialism, and pronunciation.	8 weeks	None	Final pharmacy counseling test	None	Trainee self-evaluation of progressTrainee survey of the programTrainer feedback	High counseling test pass rate.
Woodward-Kron et al, 2011^47^	Low	IMGs	N/A	Weekly 2-hour sessions with role plays, simulated examinations, video recordings, and written/verbal feedback (eg, pitch-trace diagram for stress/intonation feedback). Content: vocabulary, grammar, sound production, prosody, and discourse.	10 weeks	None	Linguist-graded video tapes on progressionAustralian Medical Council Clinical Examination pass rate	None	Trainee focus group	Improved communication skills and high clinical examination pass rate.
Koch et al, 2011^53^	Low	Nursing students	52	Web-based glossary with hyperlinks to English and Mandarin (written + audio) and quizzes. Content: teaching resources, vocabulary/medical terminology, and pronunciation.	N/A	English Language Acculturation Scale	None	None	Trainee interview	Increased confidence in communication.
Carr and DeKemel-Ichikawa, 2012^21^	Low	ESL nursing students	13	Weekly 1-hour sessions led by SLP postgraduate student clinicians. Topics: speech production and prosodic features of general American English, morphosyntax, American figurative and abstract language, and nursing terminology.	2 semesters	Language background questionnaire (the Rainbow Passage)Brief articulation test	Psycholinguistic Aspects of Foreign Accent	None	Student weekly logsStudent questionnaireFaculty feedback	Improved speech sound production and prosody. Trainees reported the program was beneficial.
Shen et al, 2012^38^ or Xu et al, 2010^58^	Low	CALD nurses	51	Weekly 2-hour sessions, taught by an SLP to reduce phonologic errors. Content: expanded from the SLP's 8-week accent reduction course regularly taught at a university.	10 weeks	Compton Phonological Assessment of Foreign Accent administered by SLP	Compton Phonological Assessment of Foreign Accent administered by SLP	None	None	Significantly less phonetic errors and linguistic errors.
Baker and Robson, 2012^49^	Low	IMGs	14	15 sessions of language training led by a language tutor (accent topics: pronunciation, stress, and intonation) + 6 sessions of consultation training led by experienced general practitioners.	6 months	Tutor-graded language skillsTutor-graded consultation skills	Tutor-graded language skillsTutor-graded consultation skills	None	Clinical supervisor questionnaireTrainee focus group	Improved language and consultation skills. Trainees reported training as helpful.
Blackburn, 2012^50^	Low	SLP students	63	1-session dialect instruction. Content: phonological and grammatical rules of African American English (AAE), AAE video clips, practice to translate standard American English (SAE) into AAE.	1 day	30-minute closed-book questionnaire (knowledge and attitude)	30-minute closed-book questionnaire (knowledge and attitude)	None	None	Improved knowledge of the AAE rules (though <50% accuracy posttest); more aware of difficulties children face in school if they don’t speak SAE.
Belay, 2013^33^	Low	ESL nurses	14	Weekly 1.5- or 2-hour sessions led by an SLP. Content: based on the Compton Pronouncing English as a Second Language program.	13 weeks	None	None	None	Trainee interview (4-8 months after training)	Increased confidence in language abilities; improved communication experiences.
Khurana and Huang, 2013^36^	Low	IMGs and IMRs	82	Weekly 90- to 120-minute classes (size: 8-14) led by accent-reduction instructors. Topics: vowels and consonants, fluency, stress, rhythm, intonation and casual speech linking, blending, contractions, and reductions.	8 to 12 weeks	Video/audio tape reviewed by 2 external assessors	Video/audio tape reviewed by 2 external assessors	Trainee self-evaluation of communication skills	Trainee self-evaluation of communication skills	Significantly improved ability to pronounce words distinctly and stress more accurately; better accuracy, intonation, fluency, speed, and volume. Increased confidence in communication.
Harvey et al, 2013^42^	Low	CALD nursing students (postgraduate)	240	6 weeks of weekly 2-hour interactive discussion sessions, + 4 weeks of role plays led by language and clinical facilitators (18 hours total). Topics: listening, speaking, and vocabulary.	10 weeks	None	Clinical practice pass rate	None	Trainee survey after 6-week discussion sessionsTrainee survey after 10 weeksTrainee survey 1 week after clinical placement completion	Similar to or higher than overall cohort pass rate for clinical practice. Increased confidence and comfort in communication and enhanced comprehension of Australian colloquial language.
Van Schaik et al, 2014^54^	Low	CALD nursing students and CALD nurses	19	8-hour practice in online course (3 20-minute practices per week). Structure: instructions, exercises, tips, and feedback. Topics: phonological and prosodic rules that govern American English pronunciation and intonation, vowels, consonants, common idioms, and figures of speech relevant in healthcare.	3 months	Online Test of English Proficiency LevelKnowledge testProficiency in Oral English Communication – Screen	Knowledge testProficiency in Oral English Communication – Screen	None	Trainee surveyTrainee exit interview	Significant gains in knowledge and verbal performance; program satisfaction.
Freysteinson et al, 2017^34^	Low	CALD health students and CALD nurses	27	Weekly 1-hour sessions taught by an SLP. Topics: role of prosody in acquisition of American English pronunciation, vowel and consonant sounds, and North American English medical terminology.	12 weeks	Self-Esteem ScalePersonal Report of Communication Apprehension-24Self-Perceived Communicative Competence	Self-Esteem ScalePersonal Report of Communication Apprehension-24Self-Perceived Communicative Competence	None	None	Significantly higher self-esteem and overall competence related to communication.

AAE, African American English; CALD, culturally and linguistically diverse; ESL, English as second language; IMGs, international medical graduates; IMRs, international medical researchers; INGs, international nursing graduates; N/A, not available; SAE, standard American English; SLP, speech language pathology/speech language pathologist.

### Synthesis of Study Results

*Participant Characteristics*. A total of 964 participants were involved in 23 studies; the other 3 studies did not specify class size/participant number but were included in this review because they reported relevant intervention descriptions, evaluation methods, and results. More than three-fourths of the study participants were health students (n=737, 76%) majoring in nursing, medicine, pharmacy, speech-language pathology, or other health sciences. The second-largest group consisted of 143 (15%) prelicensed health professionals, such as international medical graduates, international nursing graduates, and international medical researchers. In addition, 84 participants (9%) were practicing health professionals, including licensed clinicians, administrators, and clerks.

### Intervention Effects

*Beneficial Effects.* All but 2 studies demonstrated some specific benefits from accent interventions; however, the 2 inconclusive studies reported student trainees’ satisfaction with the intervention.^41,52^ Identified benefits included improvements in speech production and communication competence, improvements in academic or clinical performance, and positive perceptual changes, as summarized in Table 4. The majority of the studies (n=20, 77%) indicated program satisfaction based on trainee or trainer feedback.

**Table 4.** Beneficial Effects of Accent Interventions

Key Themes	Beneficial Effects	Studies
Speech production	Better communication clarity and/or improved communication experiences	Bosher and Smalkoski, 2002^39^Satter et al, 2005^55^San Miguel et al, 2006^45^Parkhurst, 2007^44^Seibold et al, 2007^46^Shah, 2010^37^Boughton et al, 2010^40^Cross and Smalldridge, 2011^7^Woodward-Kron et al, 2011^47^Carr and DeKemel-Ichikawa, 2012^21^Baker and Robson, 2012^49^Belay, 2013^33^Harvey et al, 2013^42^Freysteinson et al, 2017^34^
	Increased intelligibility	Parkhurst, 2007^44^Shah, 2010^37^Woodward-Kron et al, 2011^47^Carr and DeKemel-Ichikawa, 2012^21^Baker and Robson, 2012^49^Khurana and Huang, 2013^36^Van Schaik et al, 2014^54^
	Fewer phonological errors	Shah, 2010^37^Carr and DeKemel-Ichikawa, 2012^21^Shen et al, 2012^38^Baker and Robson, 2012^49^Khurana and Huang, 2013^36^Van Schaik et al, 2014^54^
Communication competence	Better listening comprehension ability	Guhde, 2003^35^Parkhurst, 2007^44^Shah, 2010^37^Baker and Robson, 2012^49^Harvey et al, 2013^42^
	Improved knowledge of colloquial language	Chur-Hansen and Barrett, 1996^51^Shah, 2010^37^Boughton et al, 2010^40^Van Schaik et al, 2014^54^
	Improved knowledge of phonological rules	Shah, 2010^37^Blackburn, 2012^50^Van Schaik et al, 2014^54^
Academic/clinical performance	Improved academic/clinical exam pass rate	Symes et al, 2002^22^Bosher and Smalkoski, 2002^39^San Miguel et al, 2006^45^Parkhurst, 2007^44^Kokkinn and Stupans, 2011^43^Woodward-Kron et al, 2011^47^Harvey et al, 2013^42^
	Higher student retention rate	Symes et al, 2002^22^
	Less clinical incidents	Yahes and Dunn, 1996^48^
	Better health survey response rate	Satter et al, 2005^55^
Perceptual changes	Increased confidence and/or self-esteem in communication	Symes et al, 2002^22^San Miguel et al, 2006^45^Boughton et al, 2010^40^Koch et al, 2011^53^Belay, 2013^33^Khurana and Huang, 2013^36^
		Harvey et al, 2013^42^Freysteinson et al, 2017^34^
	Improved collegiality	Yahes and Dunn, 1996^48^Guhde, 2003^35^Boughton et al, 2010^40^Carr and DeKemel-Ichikawa, 2012^21^Harvey et al, 2013^42^
	Improved attitude towards people with accent/dialect	Satter et al, 2005^55^Blackburn, 2012^50^
	Increased job satisfaction	Yahes and Dunn, 1996^48^

*Adverse Effects.* Adverse effects or unintended consequences were seldom discussed in the included studies. No objective measures of adverse events were reported, but 3 studies provided qualitative comments on the topic. In a study with culturally and linguistically diverse nurses, the length of the accent reduction training program (10 weeks) was reported to have fatigued some participants; the authors also observed that videotaping encounters with standardized patients added anxiety for some participants.^58^ In a language-training program for international medical graduates, disruption of normal work patterns in small departments was mentioned.^49^ The third study reporting adverse effects implemented a web-based training program for nursing students, and the author reported “two students felt marginalized and suggested that more emphasis be placed on creating opportunities for them to communicate with local students.”^53^

*Medium-Term Effects.* Most studies reported only short-term effect data collected immediately or almost immediately after completion of the intervention. The longest follow-up period was 2 semesters after the completion of a 2-semester supplementary support program for nursing students.^22^ In another study, trainees were interviewed 4 to 8 months after a 13-week training.^33^ In another study, a trainee questionnaire survey was administered 3 months after a 2-semester training.^46^ These 3 studies all reported positive trainee feedback on the intervention effects.

### Quality of the Included Studies

As shown in Table 3, all included studies were of low research quality and often had small sample sizes and few objective outcome measures, indicating a lack of generalizability and reproducibility. Only one study—a controlled before-after study—had a control group.^38^ Most studies (n=22, 85%) were case series (also known as uncontrolled longitudinal studies), which were before-after studies with no comparison group as a control. The remaining 3 studies^35,37,55^ had a case study design.

A range of objective and subjective outcome measures was used in the included studies. Notably, in every one of the 26 studies, a different set of evaluation measures was chosen or developed. Eighteen studies (69%) tested some objective measure (pretest and/or posttest); 8 (31%) reported only qualitative data. Objective posttest outcome measures used to assess intervention effects included study-specific indicators such as knowledge tests based on the content of the training program^50,54^ and standardized/established instruments such as the following:

- Australian Medical Council Clinical Examination:^59^ Strengths include being a practical clinical test and representative of the performance and knowledge expected in clinical situations.
- Comprehensive Assessment of Accentedness and Intelligibility:^57^ Tool for diagnosis and evaluation of a wide variety of speech, auditory, and nonverbal features related to accents/dialects that improves upon the Compton Phonological Assessment of Foreign Accent,^60^ the Psycholinguistic Aspects of Foreign Accent,^61^ and the Proficiency in Oral English Communication – Screen^62^ tools by addressing wider variables and supporting pretraining diagnostics.
- Compton Phonological Assessment of Foreign Accent^60^ and Psycholinguistic Aspects of Foreign Accent:^61^ Similar tools that measure speech features relevant to accents, with a focus on phonology.
- Proficiency in Oral English Communication – Screen:^62^ Measures accent-related speech features and improves upon the Compton Phonological Assessment of Foreign Accent and Psycholinguistic Aspects of Foreign Accent by addressing wider speech variables such as prosody.
- Personal Report of Communication Apprehension-24:^63^ Focuses on self-perceived comfort/apprehension with communication.
- Self-Esteem Scale:^64^ Validated instrument used to measure self-esteem.
- Self-Perceived Communicative Competence:^65^ Focuses on self-perceived communication competence.

### Role of Language or Communication Experts

Most studies included in this review involved language/communication experts—an SLP or English teacher—in the delivery of accent interventions. These experts led the training either as a sole instructor^21,33,34,36-38,45,49,50^ or as part of a multidisciplinary teaching team along with a clinical teacher.^7,22,41-44,47,48^ The latter collaborative teaching style was unanimously praised and recommended in all of the studies that used this style. ESL teachers, with understanding of linguistic complexity, are able to teach aspects of language in detail with a specific curriculum, goals, and lesson plans. SLPs, with expertise and experience in speech and language therapy, are also ideally suited to conduct regional or foreign accent improvement programs.^66^

## DISCUSSION

### Effects and Need for Accent Interventions

Despite the lack of standardized outcome measures in the studies included in this review, the current evidence suggests that accent interventions in the healthcare context can have direct benefits on communication quality: improvement in speech production, clarity, intelligibility, listening comprehension, knowledge of colloquialism and phonological rules, and reduction of phonological errors. Indirect benefits include improved academic and clinical performance and positive perceptual changes, such as improved collegiality and job satisfaction. The demonstrated range of beneficial effects provides a strong case for implementing accent-related communication improvement strategies in healthcare to support the diverse healthcare workforce serving increasingly diverse populations.

Long-term effects and adverse effects were seldom measured in the included studies. Only 3 studies reported qualitative comments that identified problems with participant fatigue and anxiety,^58^ disruption of normal clinical work,^49^ and feeling marginalized.^53^ We recommend that future studies further explore such issues and comprehensively assess the intervention impact on the participants. Documenting such unintended consequences and long-term effects is important because accent interventions are largely unregulated and free of the jurisdiction of any particular clinical practice area. In other words, no specialized license or accreditation requirements and no guiding ethical code of conduct apply to this area of practice. Accent modification services, for instance, are offered by a variety of practitioners, including licensed professionals (SLPs), ESL teachers, and nonprofessionals. Overidentification and underidentification of participants are possible in accent modification referrals. Exclusionary and discriminatory marginalization of a culturally and linguistically diverse individual, simply on the basis of an accent that differs from the local norm, is a problem of linguistic profiling.^67^ We recommend that future studies of accent interventions document the participant selection criteria and plan for rigorous assessment of both beneficial and adverse effects in the short and long term.

### Lack of Standardized Outcome Measurements

Every included study used a different set of evaluation measures, ranging from objective measures of speech production, to clinical examinations with standardized patients, to qualitative feedback. The lack of control groups and the lack of comparison from pretest to posttest using consistent objective instruments limit the strength of the current evidence base. The substantial heterogeneity in effect measures reflects a lack of standardized assessment tools in this field and a lack of methodological rigor to support generalizability and reproducibility.

The objective assessment tools that were used in some of the studies have strengths and weaknesses that require a careful examination for adoption in accent interventions. Future research that evaluates accent interventions should consider using standardized measurement tools to provide comparable findings. Moreover, none of the included studies reported findings on patient outcomes or the cost-effectiveness of the accent interventions. Future research may explore these important topics to guide practice. We recommend a comprehensive assessment of accent intervention effects to measure not only the effects on communication and intelligibility improvement but also flow-on effects, including patient satisfaction and health outcomes.

### Variability of Accent Intervention Elements

The accent interventions included in this review were all training programs for healthcare professionals or students, most of whom had a culturally and linguistically diverse/ESL background. The heterogeneity of the intervention design is evident in the great variety of program elements in terms of training content, structure, and duration. The linguistic topics in the training content varied from program to program, ranging from vowel and consonant production and stress and prosody training to higher-order language elements such as grammar, colloquialisms, idioms, small talk, formulaic expressions, paralinguistic features, medical terminology, and listening comprehension.

A needs assessment, often via trainee survey or focus group session, was conducted in a few studies before the accent intervention was implemented. Only one intervention, a case study of a one-on-one training program,^37^ was tailored to address specific speech areas based on structured pretraining diagnostics using the Comprehensive Assessment of Accentedness and Intelligibility.^57^ A few other studies recorded pretest speech performance based on tutor-graded consultation skills or established instruments such as the Compton Phonological Assessment of Foreign Accent^60^ and Proficiency in Oral English Communication – Screen.^62^ Future research may explore the feasibility and impact of using such instruments with medium or large groups of trainees to guide the customization of accent training programs to meet individual trainees’ needs.

The inconsistency of the content and procedure among accent interventions highlights a need to develop evidence-based, best-practice guidelines in this field. Laboratory research in speech sciences suggests training in vowel production,^68-70^ consonant production,^71,72^ suprasegmental features,^73-75^ auditory discrimination,^76^ and listening comprehension.^23,24,77^ Rigorous evaluation research in the healthcare setting to assess the impact of accent interventions that is based on these laboratory findings may strengthen the evidence base and contribute to the standardization of practice.

None of the included studies reported on the scalability of their accent interventions, but the 2 fully automated web-based training programs^53,54^ appeared promising, with both indicating benefits according to trainee feedback. No other technology-based interventions that particularly address the accent/dialect problem in healthcare were retrieved in the search. However, general communication assistance tools are effective in this setting. For example, a paper-based visual communication tool to assist clinician-patient interaction during counseling was found effective.^78^ Giving patients a printout of their results or a written explanation to assist during encounters with accented physicians has been suggested.^14^ Such visual assistive tools (paper-based or technology-based) might be useful in addressing communication barriers related to linguistic diversity and might be worth testing further. Future research should also gather evidence on the cost-effectiveness of accent interventions whether technology-based or not.

### Implication for Accent Intervention Practice

Accent interventions in healthcare should aim for improvement in speech production in terms of clarity and intelligibility, listening comprehension ability, knowledge of colloquialism and phonological rules, and reduction of phonological errors. Further goals of accent intervention may include better academic or clinical performance and positive perceptual change.

Accent training, ideally delivered by an SLP, may plan for curriculum content on vowel and consonant production, suprasegmental features, phonological variation, auditory discrimination, listening comprehension, vocabulary variability, nonverbal cues, and pragmatic cues (ie, social use of language). Decisions regarding other accent intervention elements may also require careful consideration in terms of the target population, intervention structure, duration, and delivery media, as well as diagnostics and assessment of trainee performance, ideally using objective tools. The rationale for each decision should be documented and reviewed posttraining to assist with the intervention evaluation. The evaluation should also identify the program impact on patient outcomes and health system outcomes, including adverse or unintended consequences in the short and long terms.

### Study Limitations

The topic of this review was limited to spoken language diversity in the healthcare setting, especially related to accent and dialect. We did not consider other aspects of communication differences, such as nonverbal linguistic diversity and other dimensions of cultural competence in healthcare. We also excluded translation, interpretation, and transcribing services provided by humans or technology. By including only the interventions that had an element to address accent- or dialect-related communication problems, we aimed to add specific knowledge regarding the efficacy of accent-targeted interventions in healthcare.

The clear heterogeneity of the accent interventions and outcome measures prevented any meaningful metaanalysis of the current data. No randomized controlled trials on accent intervention in the health setting were identified. Instead of reporting risk of bias in each study, we enhanced the review rigor via robust study inclusion criteria and study quality assessment criteria based on the literature and clinical expertise. All included studies had low quality, highlighting the need to strengthen the evidence base in the accent intervention evaluation field. We recommend implementing large randomized controlled studies that not only measure the intervention effects with standardized instruments but also collect data on cost-effectiveness.

## CONCLUSION

Despite the low research quality and lack of standardized measures in the included studies, this review establishes a sufficient evidence base to advocate for implementing accent-related communication improvement strategies in healthcare. The key benefits associated with accent interventions include improvements in speech production, communication competence, and academic/clinical performance, as well as perceptual changes. Large randomized controlled studies would be helpful to strengthen the evidence base regarding accent interventions in healthcare.

## Notes

This article meets the Accreditation Council for Graduate Medical Education and the American Board of Medical Specialties Maintenance of Certification competencies for Patient Care and Interpersonal and Communication Skills.
